# Automatic integration of numerical formats examined with frequency-tagged EEG

**DOI:** 10.1038/s41598-021-00738-0

**Published:** 2021-11-01

**Authors:** Mila Marinova, Carrie Georges, Mathieu Guillaume, Bert Reynvoet, Christine Schiltz, Amandine Van Rinsveld

**Affiliations:** 1 Institute of Cognitive Science and Assessment, Department of Behavioural and Cognitive Sciences, Faculty of Humanities, Education and Social Sciences, University of Luxembourg, 11, Porte des Sciences, 4366 Esch-Belval, Esch-sur-Alzette, Luxembourg; 2 Faculty of Psychology and Educational Sciences, KU Leuven @Kulak, Kortrijk, Belgium; 3 Brain and Cognition, KU Leuven, Leuven, Belgium; 4 Center for Research in Cognition and Neurosciences, ULB Neuroscience Institute, Université Libre de Bruxelles (ULB), B-1050 Bruxelles, Belgium; 5 Graduate School of Education, Stanford University, 505 Lasuen Mall, Stanford, CA 94305 USA

**Keywords:** Neuroscience, Psychology

## Abstract

How humans integrate and abstract numerical information across different formats is one of the most debated questions in human cognition. We addressed the neuronal signatures of the numerical integration using an EEG technique tagged at the frequency of visual stimulation. In an oddball design, participants were stimulated with standard sequences of numbers (< 5) depicted in single (digits, dots, number words) or mixed notation (dots—digits, number words—dots, digits—number words), presented at 10 Hz. Periodically, a deviant stimulus (> 5) was inserted at 1.25 Hz. We observed significant oddball amplitudes for all single notations, showing for the first time using this EEG technique, that the magnitude information is spontaneously and unintentionally abstracted, irrespectively of the numerical format. Significant amplitudes were also observed for digits—number words and number words—dots, but not for digits—dots, suggesting an automatic integration across some numerical formats. These results imply that direct and indirect neuro-cognitive links exist across the different numerical formats.

## Introduction

The question of where the meaning of numbers comes from challenged both philosophers and scientists for many centuries. In the past decades, research on numerical cognition has demonstrated that since early infancy, humans, similar to other animals, are endowed with the ability to perceive the approximate number of items in a set in a fast and effortless manner^1^. Unlike any other animal, however, humans are capable of learning symbolic number notations (e.g., digits, number words), allowing them to manipulate numbers exactly. Yet, it remains to be determined how symbolic and non-symbolic number processing are integrated with each other and whether this happens automatically (i.e., unintentionally). Assessing the automaticity of this integration constitutes a window into the building block of abstract human thinking.

### The relation between symbolic and non-symbolic numbers

Numerical information can be conveyed non-symbolically as numerosities (i.e., sets of items) and symbolically as digits and number words. The exact relation between these three numerical notations has been extensively debated^2–5^. On the one side, it has been argued that the symbolic and non-symbolic numbers share common mental representations, grounding in the so-called Approximate Number Sense^1,4,6–9^. An observation commonly interpreted as evidence in this regard, comes from neuro-cognitive studies, demonstrating that shared neuronal populations for symbolic numbers and non-symbolic numbers exist within parietal brain regions, more specifically along the Intra Parietal Sulcus (IPS)^4,8–14^. For instance, in their seminal study, Piazza and colleagues^14^ used an fMRI adaptation paradigm, in which participants passively viewed either small or large dot arrays, symbolic numbers (digits, number words), or a mix of symbolic numbers and dots. After the period of adaptation, a novel stimulus was presented, which could differ in its notation or in number. The results showed a cross-notational neuronal distance effect: a greater distance between numbers makes them more discriminable. That is, irrespectively of the notation, a sustained decrease in the BOLD signal within the IPS was observed when the novel stimulus was numerically close to the adapted one, but not when the numerical distance was large. These results suggested the existence of a tight automatic link (i.e., integration) between symbolic numbers and numerosities; and were further interpreted as evidence that the IPS is the brain locus of the abstract representation of numerical magnitude regardless of their format.

On the other hand, the neuro-cognitive integration between the symbolic and non-symbolic numbers has also been seriously questioned^2,3,15^ in favour of the existence of notation-dependent neuronal populations within the parietal cortex^16,17^. For instance, already in 2011, Cohen Kadosh^18^ and colleagues showed that no cross-notational adaptation occurred within the parietal cortex. Furthermore, it was demonstrated that the neuronal populations within the parietal regions were sensitive to the format change rather than to the changes in the number^19,20^. Similarly, using classifiers to identify the activation pattern of the IPS for different numerical notations (i.e., digits, dots, and dots—digit), Bulthé and colleagues^21,22^ demonstrated that a neuronal distance effect was present only in the non-symbolic number condition, but not in the symbolic condition. Moreover, the results showed that although the IPS was active for both symbolic and non-symbolic notations, the pattern of activation was qualitatively distinct—above chance level classification accuracies were observed for numerosities only (≈77%). For symbolic and for cross-notation conditions (i.e., digits—dots), the classification patterns were substantially poorer (< 57%). According to the authors, these findings indicate that, unlike dot patterns, the numerical information conveyed by the digits is possibly processed and retrieved from regions outside the IPS. Taken together, these studies are difficult to reconcile with the existence of a common abstract representation of number and rather indicate that symbolic and non-symbolic numerical processing do not share the same representation in the brain.

### The automaticity of the integration between symbolic and non-symbolic numbers

The discrepancies observed in the literature might be related to differences in the stimulus characteristics (e.g., number range and numerical correspondence between formats, see also Liu et al.^23,24^) and experimental tasks. Indeed, the majority of the studies showing evidence for format interdependence have employed explicit tasks (i.e., required participants to make an intentional numerical decision), while implicit measures are rarely used. Explicit task settings might urge participants to intentionally relate (and possibly integrate) the numerical formats^20^, while this would not be the case when participants are not required to make a numerical decision. Consequently making use of implicit paradigms (e.g., passive viewing) —where the numerical representation is probed per se, independently of any decisional strategies^20,25^, might be better suited to provide an objective (i.e., free of any intentional confounds) measure of the integration across numerical formats^26–28^. Two EEG studies used such implicit measures to examine the integration across numerical formats^23,29^. Their findings, however, are somewhat contradicting.

Van Hoogmoed and Kroesbergen^29^ used a passive-viewing ERP (event-related potential) design and presented participants with numbers depicted as digits, dots or dots and digits. The results showed significant differences between the ERPs for dots—dots vs digits—dots, both early and late in the visual stream, possibly suggesting that different cognitive processes underlie the performance in the two conditions. Liu et al.^23^, on the other hand, found evidence for an automatic (i.e., spontaneous and unintentional) integration between digits and numerosities (see also^24,30^). Here, participants were presented with double-digit numbers superimposed over dots arrays, which could either match (i.e., “20” over twenty dots) or mismatch (i.e., “20” over fifteen dots) in terms of their perceived numerical value. Liu et al.^23^ found significantly higher amplitudes (N1 and P2p) for the match trials. These results were interpreted as evidence that digits and dots are integrated spontaneously and unintentionally.

The contradicting results and interpretations possibly stem from the methodological parameters of these studies. For instance, both studies made use of large sets of approximate numbers (i.e., > 10) which are known to form less stable mappings with symbolic numbers than small numbers^31–33^. Consequently, using small numbers, where stable numeral-to-numerosity mappings are observed, might be better suited to investigate the integration between symbolic and non-symbolic numbers. In addition, small symbolic and non-symbolic numbers (e.g., 1 to 9) are more frequently encountered and are thus represented with higher precision, compared to the large numbers (e.g., > 15^34^), allowing to compare participant’s performance across the two numerical formats directly. Finally, both Van Hoogmoed and Kroesbergen^29^ and Liu et al.^23^ used only digits and dots, thus leaving the question about how the other symbolic notation—number words is integrated unaddressed. Against this background, it becomes apparent that further research investigating the automaticity of the integration between all numerical notations is needed. In the current study, we considered the abovementioned methodological considerations and used frequency-tagging EEG to investigate the question of number format integration objectively.

### Frequency-tagged EEG

This EEG technique consists of measuring steady-state visual evoked potentials (SSVEP) tagged at the frequency of a fast periodic visual stimulation^35^. More specifically, the fast periodic visual stimulation consisted of a rapid presentation of standard stimuli (i.e., F = 10 Hz), among which a deviant stimulus (i.e., oddball) appeared every 8th item. If the system discriminates between standard and deviant stimuli, we should record a response at the oddball frequency *F*/*n* (i.e., 1.25 Hz). This approach has the advantage of being free of any explicit task instructions and thus provides an objective measurement index. In addition, the SSVEP paradigm is of a higher signal-to-noise ratio compared to the more traditional measures in numerical cognition such as ERP^35^. Previous research demonstrated that SSVEP oddball paradigms are suited to measure both low-level visual processing^35^, as well as higher cognitive mechanisms such as face discrimination^36,37^, language processing^38^ and semantic categorization^39^. This technique has been successfully used for investigating numerosity processing^40–44^. So far, only one study has employed SSVEP to track symbolic number discrimination, indicating that people automatically process the magnitude of symbolic numbers^45^. Taken together, these results demonstrate that frequency-tagged EEG is sensitive to symbolic and non-symbolic number processing.

**Figure 1:**
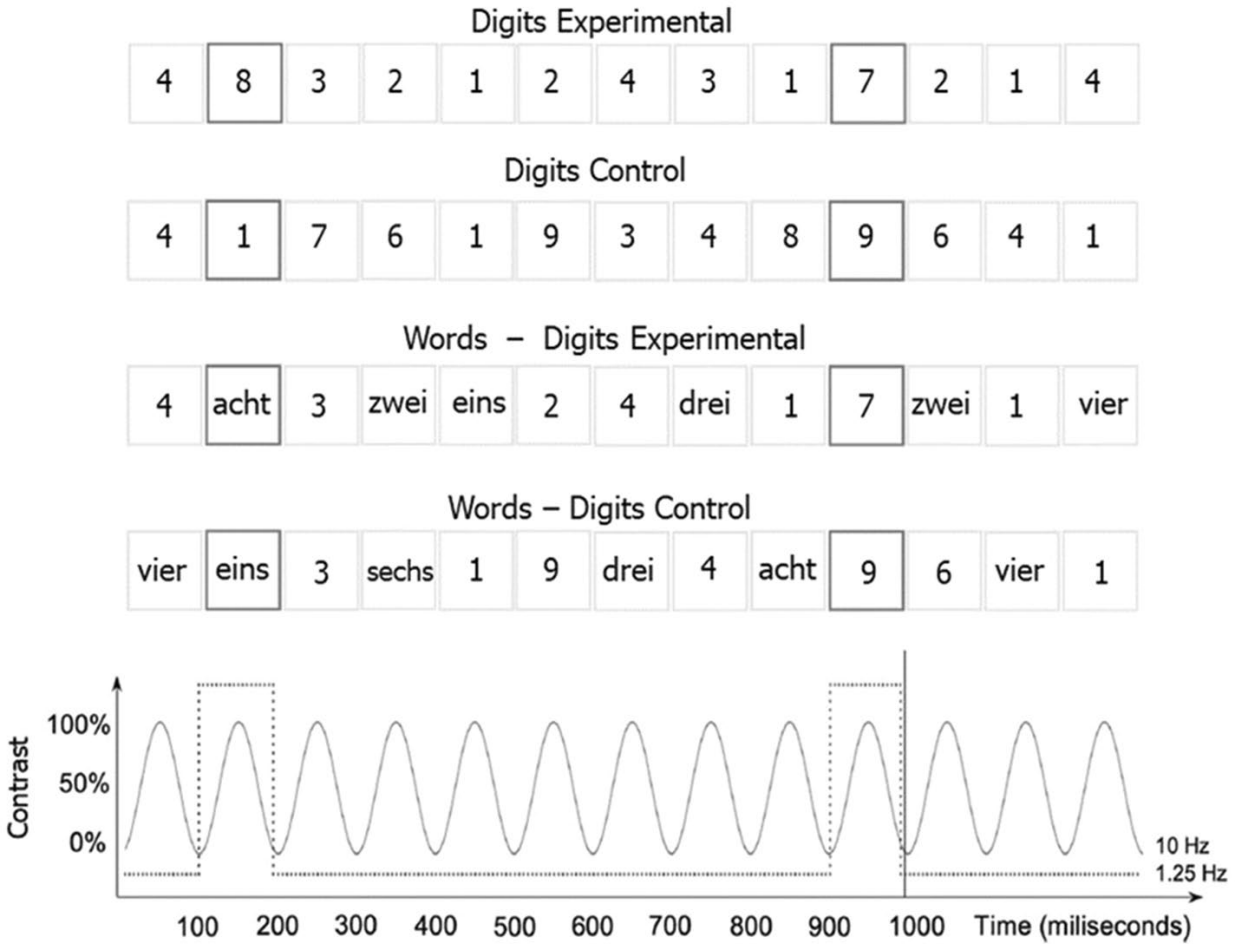
Visual depiction of (some of) the single and mixed notation conditions of the Frequency-tagged EEG oddball design. Sequences were presented for 48 s in both experimental and control conditions. For the experimental conditions, participants saw either single notations sequences (digits, words, or dots), or mixed notation sequences conditions (digits—words, words—dots, digits—dots). Irrespectively of the notation, numbers smaller than five were presented as base stimuli at the frequency of 10 Hz, and every 8th item, a number larger than 5 was inserted at the oddball frequency of 1.25 Hz. In the corresponding control conditions, the categories (base vs. oddball stimuli) were assigned randomly. The bottom panel illustrates the onset and the offset of each stimulus following the sinusoidal contrast modulation from a 0 to 100% contrast. The number words were presented in German, which translated into English as follows: “eins”—“one”, “zwei”—“two”, “drei”—“three”, “vier”—“four”, “fünf”—“five” (not included in the stimulus list), “sechs”—“six”, “sieben”—“seven”, “acht”—“eight”, “neun”—“nine”.

In the current study, we applied this technique for the first time to the integration of magnitude information across numerical formats: we compared implicit, rapid 1–9 magnitude discrimination in streams of digits, number words, and dots (single notation) to streams of mixed formats—digits-words, dots-digits, words-dots (mixed notation, see Fig. 1). Participants were presented with sequences consisting of numbers smaller than 5 (1–2–3–4) as standard and numbers larger than 5 (6–7–8–9) as deviant (i.e., oddball) stimuli in all conditions. Additionally, a control condition with a random assignation of numbers to the standard and deviant categories, without any rule over the magnitude was also presented. If the magnitude is spontaneously and unintentionally discriminated irrespectively of the notation (symbolic vs non-symbolic), we expect to observe EEG responses tagged at the frequency of the oddball in the three single notation formats (H1). Furthermore, if digits, words, and dots are spontaneously and unintentionally integrated with each other due to one common mental representation of number, we expect mixed notation sequences to yield similar oddball responses. In contrast, if symbolic numbers and numerosities are not automatically integrated, and thus rather processed by distinct mechanisms, we expect different EEG oddball signatures across the mixed notation sequences (H2).

## Method

### Participants

Participants were recruited via an online subscription system. Nineteen undergraduate students aged between 18 and 25 years (*M*_*age*_ = 20.68 years, *SD* = 2.34, 2 males) participated in an exchange of a 25-euros gift voucher. The experimental protocol and the methods were in accordance with the APA ethical standards and with the institutional guidelines and regulations provided by the Ethics Review Panel at the University of Luxembourg. The Ethics Review Panel at the University of Luxembourg approved the current study, its methodology, and implementation (file number ERP 19–025). Prior to participation, written informed consent was sought from each participant. All participants had a normal or corrected-to-normal vision, with no history of neurological or learning disorders. No participants were excluded due to excessive noise in EEG data or due to not looking at the screen during the presentation of the stimuli.

### Materials, stimuli, and procedure

MATLAB (The MathWorks) with Psychophysics Toolbox extension was used for the presentation of the stimuli. Participants were comfortably seated at 1 m away from the computer screen (24 inches LED monitor, 1280 × 1024 px resolution, 100 Hz refresh rate, 1 ms response time). Each participant was presented with the following number notation conditions: (1) digits, (2) dots, (3) words, (4) digits and words, (5) dots and digits, and (6) number words and dots in a frequency-tagged EEG oddball design. For each condition, there was an experimental and a corresponding control condition, resulting in a total of 12 conditions. In the experimental condition, the frequent stimuli were numbers smaller than 5 (i.e., 1,2,3,4) and the oddball stimuli were numbers larger than 5 (i.e., 6,7,8,9) (see Fig. 1). That is, in the single format conditions, both the standard and the deviant stimuli were of the same format with the only difference being whether they are smaller (base-rate) or larger (oddball) than five. In the mixed notation conditions, the standard stimuli contained numbers smaller than five, presented in both formats (e.g., digits, and numbers words presented randomly and non-periodically), and the deviant stimuli were numbers larger than five, presented in both formats (again randomly and non-periodically varied). For the corresponding control conditions, the presentation of the numbers was completely random. That is, there was no rule which numbers will be presented at 10 Hz and which at 1.25 Hz. The experimental and corresponding control conditions per notation were repeated four times each, resulting in a total of 48 repetitions per participant. The experimental and corresponding control conditions were presented in blocks per notation condition (e.g., four repetitions for digit experimental, followed by four repetitions for digit control). The order of the blocks and the order of the conditions within the blocks was counterbalanced across participants in a Latin-square design.

To control for perceptual habituation to the visual properties of the stimuli, stochastic physical variations were introduced during the presentation of the stimuli^35^. Consequently, the font type and size used for the digits and number words was randomly varied across stimuli (i.e., Arial, Times New Roman, Cambria, and Calibri). For the digits, the size varied between 80 px and 120 pixels with a mean value of 100 pixels ( ≈ 1.85° vertical and ≈ 1.14° horizontal visual angle^45^). To make sure that the number words were not appearing outside the participants’ central fixation point and can be read without saccade, their size was on average 70% of the digits’ size (i.e., between 50 and 90 px, i.e., ≈ 0.92° to 1.66° vertical, and no more than ≈ 5.50° horizontal visual angle). The stimuli were presented centrally on the screen. Dot pictures were generated with the NASCO software^46^, controlling for non-symbolic visual cues (i.e., the total area occupied, item size, convex hull, and mean occupancy). All stimuli were presented at a base rate of 10 Hz (i.e., 10 stimuli per second), in which a rare stimulus was inserted at a rate of 1.25 Hz (i.e., every 8th stimulus) (see Fig. 1). Stimuli appeared on the screen following a sinusoidal contrast modulation from 0 to 100%. For each notation condition, the stimuli were presented in sequences of 48 s –44 s of stimulation, and 2 s of fade-in and fade-out, the latter being further excluded from the analyses.

### EEG data acquisition

The EEG signal was acquired with a 64-channel Biosemi ActiveTwo system (BioSemi B. V., Amsterdam, The Netherlands). The location of the electrodes on the gap was according to the standard 10–20 system (for details see http://www.biosemi.com). The Common Mode Sense (CMS) and the Driven Right Leg (DRL) served as the reference electrodes. The electrode offset was held below 40 microvolts (μV).

### Data preprocessing

Data were downsampled from 2048 to 512 Hz to decrease the computational load of further analyses. Further analyses were performed using *Letswave 6* software (http://nocions.webnode.com/letswave). First, data were filtered (band-pass cut off 0.1- 100 Hz) and re-referenced to the common average. Noisy channels were interpolated using three of the closest neighbouring electrodes. This procedure was only done in one subject where P5 was interpolated on P3, P7, and PO3.

Second, building upon prior studies using frequency-tagged responses to digits and dots^41,45^, we expected to record responses over the posterior scalp and thus grouped electrodes by pooling them in three relevant regions of interest (ROI)/electrode sides: Medial Occipital (O1, Iz, Oz, O2), Left Occipito-Parietal (P5, P7, P9, PO7), and finally Right Occipito-Parietal (P6, P8, P10, PO8). According to the standard Biosemi 64-channel system, the averaged electrodes were the following: Medial Occipital—A27, A28, A29, B32; Left Occipito-Parietal: A22, A23, A24, A25; and Right Occipito-Parietal: B27, B28, B29, B30 (see https://www.biosemi.com). Third, the EEG signal was segmented in 44 s epochs containing 440 stimulus onsets and 22,528 data points (i.e., fade-in and fade-out seconds were not included). Third, we averaged the four repetitions of each condition for each participant. Finally, Fast Fourier Transformation (FFT) was applied to the data.

### EEG analyses

**Figure 2:**
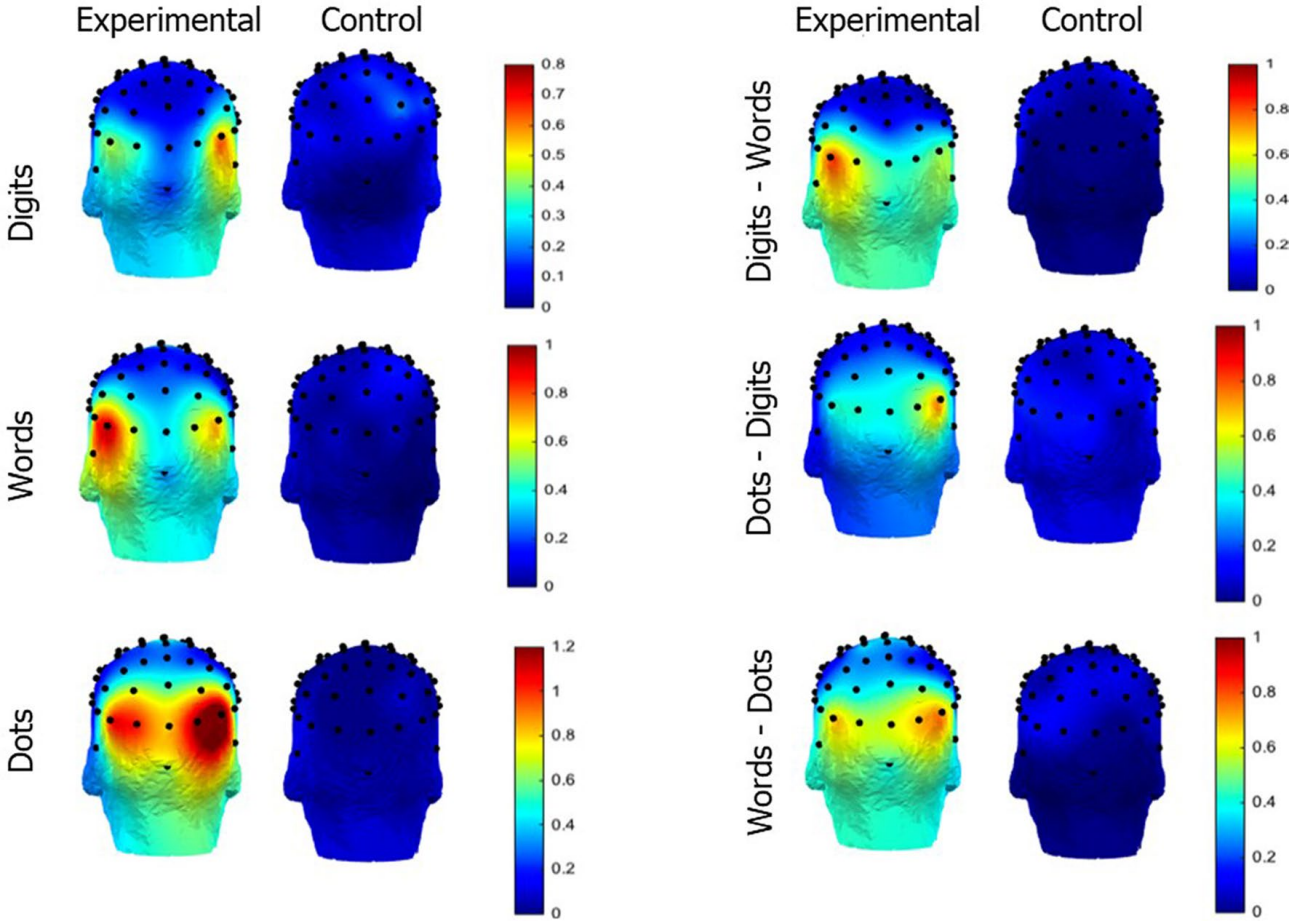
Scalp topographies of the SBAs (in microvolts) depicted per condition and notation.

First, to assess possible differences in the oddball responses across the number notation conditions, we computed the Sums of the Baseline Corrected Amplitudes (SBAs) across the oddball frequency and its seven harmonics (i.e., 1.25 Hz, 2.5 Hz, 3.75 Hz, 5 Hz, 6.75 Hz, 7.5 Hz, and 8.75 Hz; for a similar method see^40,41,45^) by subtracting the mean amplitude of the twenty surrounding bins (10 on each side) to each bin, excluding the immediately adjacent bins and two most extreme bins^35,36^. We then averaged the data across participants and per notation condition. Figure 2 shows the whole scalp topographies of the seven harmonics. The amplitude spectra expressed as baseline-corrected amplitudes for each notation condition and for each electrode side is depicted in Fig.S1 in the supplementary material.

Second, to assess the statistical significance of the oddball responses, we used z-scores. These z-scores were obtained by first segmenting the individual FFT data around the response of interest and its harmonics (e.g., 1.25 Hz, 2.5 Hz, 3.75 Hz, 5 Hz, 6.75 Hz, 7.5 Hz, and 8.75 Hz) into successive bins (12 on each side) and then summing across the frequency spectra. The same procedure was applied to the base responses (i.e., 10 Hz), however, here summing across the spectra was not needed. After that, for both oddball and base responses, we applied z-score transformations to each bin as a function of its twenty surrounding bins (10 on each size, see^41^. Conventionally, z-scores above 1.64 (i.e., *p* < 0.05 one-tailed; hypothesis tested signal < noise) indicate significant brain responses to the oddball category.

## Results

The current study’s data set and statistical output are freely available on the Open Science Framework (https://osf.io/5wf8u/). Averaged z-scores per notation, condition, and electrode side (i.e., ROI) for both the oddball and base amplitudes are depicted in Table 1. The results clearly demonstrated that all conditions yielded very strong base rated responses, indicating that the stimulation was successful and participants attended the presentation. Furthermore, concerning the oddball responses, the experimental conditions tended to yield significant oddball responses, while no significant oddballs were present in the control conditions. The experimental conditions for dots, words, digits—words, and words– dots notations yielded significant responses across (almost) all electrode sides, leading to an overall strong and significant response. The experimental conditions for digits and dots—digits, yielded significant amplitudes in only one electrode side, leading to an overall weaker and not significant amplitudes across all electrode sides. To examine the differences between notations and across the different electrode sides, the z-scores were submitted to a series of repeated measures ANOVAs. Whenever the assumption of sphericity was violated, Greenhouse–Geisser correction was applied. Data were also analysed in Bayesian statistical frameworks. We report the Bayes factors (BF)—or log(BF) in case the BF values are too large to interpret^47,48^. The BF is the ratio of the likelihood of the alternative hypothesis and the likelihood of the null hypothesis. For statistical analyses, involving a larger number of factors such as repeated-measures ANOVA, it is recommended to report the BF_Inclusion_^48^. Conventionally, the evidence provided by the BF values is categorized as “anecdotal” (for values between < 1 and 3), “moderate” (for values between 3 and 10), “strong” ( for values between 10 and 30), “very strong” (for values between 30 and 100), and “extreme” (for values > 100)^49^. Although Bayesian and classical analyses usually converge in their results, differences are possible especially concerning the interactions between factors^51^. Given that the underlying sources of these discrepancies are yet to be clarified, in case of differences, we prefer to base the interpretation of our findings on the classical analyses. To obtain both classical and Bayesian results, we used JASP statistical package v0.14 (https://jasp-stats.org/).

**Table 1:** Averaged z-scores (with the corresponding standard error (SE) of the mean) for the oddball and base frequencies, depicted per condition, notation, and electrode side. Significant z-scores (> 1.64) are marked with *. Control conditions are in italics.

Notation		EEG amplitudes (z-scores)			
Electrode side		Left occipito-parietal	Right occipito-parietal	Medial occipital	Overall
Digits	Oddball	1.71* (0.51)	2.46* (0.58)	1.00 (0.43)	1.72* (0.46)
		*−0.10 (0.30)*	*−0.18 (0.29)*	*−0.17 (0.26)*	*−0.15 (0.24)*
	Base	7.02* (1.05)	6.20* (1.05)	9.56* (1.24)	7.59* (0.78)
		7.25* (0.81)	7.87* (0.99)	10.35*(1.49)	8.49* (0.73)
Words	Oddball	3.47* (0.61)	1.82* (0.49)	1.72* (0.45)	2.34* (0.43)
		*−0.07 (0.32)*	*−0.32 (0.30)*	*−0.13 (0.30)*	*−0.17 (0.27)*
	Base	7.21* (1.63)	11.35* (1.50)	11.50* (1.54)	10.02* (1.13)
		8.50* (1.30)	11.06* (1.98)	13.48* (1.92)	11.01* (1.39)
Dots	Oddball	2.35* (0.46)	3.97* (0.84)	3.54* (0.52)	3.29* (0.50)
		*0.34 (0.20)*	*−0.02 (0.20)*	*−0.23 (0.27)*	*0.03 (0.14)*
	Base	2.73* (0.61)	2.16* (0.46)	4.47* (0.81)	3.12* (0.40)
		3.32* (0.52)	3.40* (0.64)	6.11* (1.18)	4.28* (0.57)
Digits-words	Oddball	2.90* (0.62)	1.94* (0.33)	1.91* (0.35)	2.25* (0.36)
		*−0.39 (0.22)*	*−0.41 (0.29)*	*−0.87 (0.30)*	*−0.56 (0.21)*
	Base	7.29* (0.91)	10.42* (1.80)	9.56* (2.01)	9.09* (1.12)
		*8.00 (1.20)*	9.60* (1.61)	8.28* (1.41)	8.63* (1.00)
Dots-digits	Oddball	1.18 (0.49)	1.71* (0.49)	1.35 (0.46)	1.41 (0.40)
		*0.31 (0.28)*	*0.23 (0.34)*	*0.13 (0.29)*	*0.22 (0.23)*
	Base	5.35* (0.69)	6.44* (1.01)	8.32* (1.54)	6.70* (0.83)
		7.41* (1.14)	7.39* (1.01)	10.07* (1.86)	8.29* (1.02)
Words-dots	Oddball	2.34* (0.33)	2.30* (0.53)	2.68* (0.51)	2.44* (0.39)
		*−0.11 (0.25)*	*−0.30 (0.32)*	*−0.62 (0.27)*	*−0.34 (0.23)*
	Base	6.08* (0.98)	8.22*(1.11)	8.25* (1.35)	7.52* (0.92)
		6.58* (1.02)	8.24* (1.27)	7.58* (1.00)	7.47* (0.69)

### Single notation conditions

#### Base rate (i.e., 10 Hz) response analysis

The z-scores were submitted to a 2 × 3 × 3 repeated-measures (Bayesian) ANOVA on the single format sequences, with condition (2 levels: experimental vs control), notation (3 levels: digits vs words vs dots) and electrode side (3 levels: LOP vs ROP vs MO) as within-subject factors.

#### Main effects

There was a main effect of condition, *F*(1, 18) = 18.08, *p* < 0.001, *η*_*p*_^2^ = 0.50, with control conditions, yielding overall slightly stronger base response than the experimental conditions, *p*_*bonf*_ < 0.001, *d* = 0.98. The presence of this main effect, however, was not supported by the Bayesian analysis, BF_Inc_ = 0.29. There was also a main effect of notation, *F*(1.48, 26.55) = 23.24, *pGG* < 0.001, *η*_*p*_^2^ = 0.56, BF_Inc_ > 100—the number words yielded somewhat higher base amplitudes compared to both digits, *p*_*bonf*_ = 0.06, *d* = 0.56, BF_10_ = 23.87, and dots, *p*_*bonf*_ < 0.001, *d* = 1.54, BF_10_ > 100. The base amplitude for digits was also higher than for dots, *p*_*bonf*_ < 0.001, *d* = 0.98, BF_10_ > 100. There was also a main effect of electrode side, *F*(2, 36) = 6.30, *p* = 0.004, *η*_*p*_^2^ = 0.26, BF_Inc_ > 100, yielding stronger base amplitudes for MO electrode side, compared to LOP, *p*_*bonf*_ = 0.004, *d* = 0.79, BF_10_ > 100, but not ROP sides, *p*_*bonf*_ = 0.07, *d* = 0.55, although Bayesian post-hoc comparisons showed some evidence for this difference, BF_10_ = 67.20. The ROP and LOP sides did not differ from one another, *p*_*bonf*_ = 0.87, *d* = 0.25, BF_10_ = 0.39.

#### First-order interactions

None of the first order interactions were significant: condition × notation, *F*(2, 36) = 0.03 *p* = 0.98, *η*_*p*_^2^ < 0.01, BF_Incl_ < 1; condition × electrode side, *F*(2, 36) = 0.72 *p* = 0.49, *η*_*p*_^2^ = 0.04, BF_Incl_ < 1; notation × electrode side, *F*(2.59, 46.66) = 1.53 *pGG* = 0.22, *η*_*p*_^2^ = 0.08, BF_Incl_ < 1.

#### Second-order interactions

The interaction between condition × notation × electrode side, was not significant either, *F*(4, 72) = 1.10, *p* = 0.36, *η*_*p*_^2^ = 0.06, BF_Incl_ < 1. Despite that the interaction was not significant, for the sake of completeness and to make sure that no subtle differences were present at the level of the notations, we report the post-hoc comparisons (Bonferroni correction applied) per notation, condition, and electrode side. For the digits notation, there were no significant differences between control and experimental base frequency amplitudes for neither of the electrode sites: LOP, *p*_*bonf*_ = 1.00, BF_10_ = 0.25, MO, *p*_*bonf*_ = 1.00, BF_10_ = 0.29, ROP, *p*_*bonf*_ = 1.00, BF_10_ = 1.47. This was also the case for the number words notation: LOP, *p*_*bonf*_ = 1.00, BF_10_ = 0.64, MO, *p*_*bonf*_ = 1.00, BF_10_ = 0.47 ROP, *p*_*bonf*_ = 1.00, BF_10_ = 0.24. And finally, the same results were also obtained for the dots notation: LOP, *p*_*bonf*_ = 1.00, BF_10_ = 0.36, MO, *p*_*bonf*_ = 1.00, BF_10_ = 1.41, ROP, *p*_*bonf*_ = 1.00, BF_10_ = 4.23. In sum, despite the presence of main effects, overall, we found no evidence that base-frequency responses differed between experimental and control conditions across notations and electrode sites.

#### Oddball (i.e., 1.25 Hz) response analysis

The z-scores were submitted to a 2 × 3 × 3 repeated-measures (Bayesian) ANOVA on the single format sequences, with condition (2 levels: experimental vs control), notation (3 levels: digits vs words vs dots) and electrode side (3 levels: LOP vs ROP vs MO) as within-subject factors.

#### Main effects

There was main effect of condition, *F*(1, 18) = 45.98, *p* < 0.001, *η*_*p*_^2^ = 0.72, BF_Incl_ > 100, yielding higher amplitudes for experimental than control condition, *p*_*bonf*_ < 0.001, *d* = 1.56, BF_10_ > 100. The main effect of notation was also significant, *F*(2, 36) = 6.53, *p* = 0.004, *η*_*p*_^2^ = 0.27, BF_Incl_ = 32.32—the dots yielded somewhat higher amplitudes compared to digits, *p*_*bonf*_ = 0.003, *d* = 0.82, BF_10_ > 100, but not compared to number words, *p*_*bonf*_ = 0.07, *d* = 0.54, BF_10_ = 1.42. The oddball amplitudes for digits and number words did not differ from one another, *p*_*bonf*_ = 0.71, *d* = −0.26, BF_10_ < 1. The main effect of electrode side was not significant, *F*(2, 36) = 1.85, *p* = 0.17, *η*_*p*_^2^ = 0.09, BF_Incl_ < 1.

#### First-order interactions

There was a notation × electrode side interaction, *F*(4, 72) = 6.23, *p* < 0.001, *η*_*p*_^2^ = 0.26, BF_Incl_ = 6.45. The condition × notation interaction was not significant, *F*(2, 36) = 2.46, *p* = 0.10, *η*_*p*_^2^ = 0.12, BF_Incl_ < 1, and neither was the condition × electrode side interaction, *F*(1.52, 27.35) = 1.37, *pGG* = 0.27, *η*_*p*_^2^ = 0.07, BF_Incl_ < 1.

#### Second-order interactions

**Figure 3:**
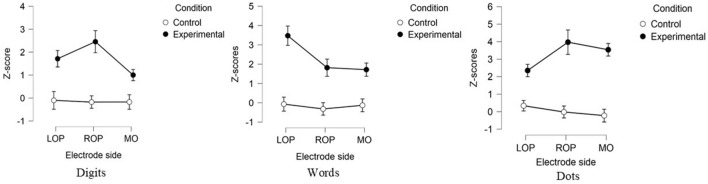
Z-scores for the single notation trials—digits, words, and dots, depicted as a function of the condition (control vs experimental) and electrode side. LOP = Left Occipito-Parietal, ROP = Right-Occipito Parietal, MO = Medial-Occipital.Vertical bars denote Standard Errors (SE).

Finally, although Bayesian ANOVA did not provide conclusive evidence for the presence of the three-way interaction condition × notation × electrode side, BF_Incl_ < 1, the classical ANOVA showed that this interaction was significant, *F*(2.21, 39.84) = 7.05, *pGG* = 0.002, *η*_*p*_^2^ = 0.28 (Fig. 3). Therefore, we performed post hoc ANOVAs per notation with condition (2 levels) and electrode side (3 levels) as within-subject factors to disentangle this interaction.

For the digits notation, there was a main effect of condition, *F*(1, 18) = 12.84, *p* = 0.002, *η*_*p*_^2^ = 0.42, with the experimental condition yielding stronger oddball amplitudes than the control condition, *p*_bonf_ = 0.002, *d* = 0.82. There was a main effect of electrode side, *F*(2, 36) = 5.12, *p* = 0.011, *η*_*p*_^2^ = 0.22, indicating stronger amplitudes for the ROP side compared to MO, *p*_bonf_ = 0.009, *d* = 0.73, but not compared to the LOP side, *p*_bonf_ = 0.45, *d* = 0.34. The LOP and MO sides did not differ from one another, *p*_bonf_ = 0.28, *d* = 0.40. There was also a condition × electrode side interaction, *F*(2, 36) = 4.99, *p* = 0.012, *η*_*p*_^2^ = 0.22. Post hoc comparisons (Bonferroni correction applied) showed that the experimental condition yielded significantly stronger peaks than the control condition only in ROP side, *p*_bonf_ = 0.002, but not in the LOP, *p*_bonf _ = 0.07, and the MO sides, *p*_bonf_ = 0.84.

For the words notation, there was a main effect of condition, *F*(1, 18) = 22.12, *p* < 0.001, *η*_*p*_^2^ = 0.55, suggesting that the experimental condition yielded higher oddballs than the control condition, *p*_bonf_ < 0.001, *d* = 1.08. There was also a main effect of electrode side, *F*(1.48, 26.55) = 7.21, *pGG* = 0.006, *η*_*p*_^2^ = 0.29, suggesting higher amplitudes for LOP side compared to both ROP, *p*_bonf_ = 0.006, *d* = 0.77, and MO sides, *p*_bonf_ = 0.008, *d* = 0.74. The ROP and MO electrode sides did not differ from each other, *p*_bonf_ = 1.00, *d* = −0.03. There was also a condition × electrode side interaction, *F*(2, 36) = 4.76, *p* = 0.02, *η*_*p*_^2^ = 0.21. Post hoc comparisons (Bonferroni correction applied) showed that experimental condition yielded significantly higher peaks than the control condition within the LOP, *p*_bonf_ < 0.001, and the ROP, *p*_bonf_ = 0.03, electrode sides, but not in the MO side, *p*_bonf_ = 0.09. Furthermore, the experimental condition within the LOP side yielded higher amplitudes then the experimental condition in ROP side, *p*_bonf_ = 0.002. This was not the case for the control condition comparisons, *p*_bonf_ = 1.00.

For the dots notation, there was a main effect of condition, *F*(1, 18) = 41.44, *p* < 0.001, *η*_*p*_^2^ = 0.70, yielding higher amplitudes for the experimental than for the control condition, *p*_bonf_ < 0.001, *d* = 1.48. There was no main effect of electrode side, *F*(2, 36) = 1.67, *p* = 0.202, *η*_*p*_^2^ = 0.09, but there was a condition × electrode side interaction, *F*(1.37, 24.66) = 4.19, *pGG* = 0.04, *η*_*p*_^2^ = 0.19. Post hoc comparisons showed that the experimental condition yielded higher amplitides than the control condition, in ROP, *p*_bonf_ < 0.001, and MO electrode sides, *p*_bonf_ < 0.001, but not in the LOP, *p*_bonf_ = 0.06. The amplitudes for the experimental condition within both ROP and MO, were significantly higher than the amplitude for LOP, all *ps*_bonf_ < 0.001. The amplitudes across ROP and MO did not differ either in the experimental or in the control conditions, *p*_bonf_ = 1.00.

### Mixed notation conditions

Base rate (i.e., 10 Hz) response analysis. Similarly, to the single notation conditions, we first analysed the z-scores for base frequency amplitudes in the mixed notation conditions. We performed a condition (2 levels) by mixed format (3 levels) by electrode side (3 levels) repeated-measures (Bayesian) ANOVA.

#### Main effects

No main effects were significant for condition, *F*(1, 18) = 0.99, *p* = 0.33, *η*_*p*_^2^ = 0.05, BF_Incl_ < 1, notation, *F*(2, 36) = 1.64, *p* = 0.21, *η*_*p*_^2^ = 0.08, BF_Incl_ < 1, or electrode side, *F*(1, 18) = 1.29, *p* = 0.29, *η*_*p*_^2^ = 0.07, BF_Incl_ < 1.

#### First-order interactions

There were no significant first order interactions for condition × notation, *F*(2, 36) = 1.86, *p* = 0.17, *η*_*p*_^2^ = 0.09, BF_Incl_ < 1, condition × electrode side, *F*(2, 36) = 2.20, *p* = 0.13, *η*_*p*_^2^ = 0.11, BF_Incl_ < 1, or notation × electrode side, *F*(4, 72) = 1.51, *p* = 0.21, *η*_*p*_^2^ = 0.08, BF_Incl_ < 1.

#### Second-order interactions

The interaction between condition, notation, and electrode side was not significant, *F*(4, 72) = 0.44, *p* = 0.78, *η*_*p*_^2^ = 0.02, BF_Incl_ < 1, altogether providing no evidence that the base-frequency amplitudes differed across the conditions. Again, for completeness, we report the post-hoc comparisons per notation. For the digits-words, the control and experimental base frequency amplitudes did not differ across the electrode sides: LOP, *p*_*bonf*_ = 1.00, BF_10_ = 0.39, MO, BF_10_ = 0.42, *p*_*bonf*_ = 1.00, ROP, *p*_*bonf*_ = 1.00, BF_10_ = 0.29. The results were identical for the dots-digits, LOP, *p*_*bonf*_ = 1.00, BF_10_ = 0.83, MO, *p*_*bonf*_ = 1.00, BF_10_ = 0.77, ROP, *p*_*bonf*_ = 1.00, BF_10_ = 0.42, and the words—dots conditions, LOP, *p*_*bonf*_ = 1.00, BF_10_ = 0.28, MO, *p*_*bonf*_ = 1.00, BF_10_ = 0.32, ROP, *p*_*bonf*_ = 1.00, BF_10_ = 0.24. In sum, similarly, to the single notation trials, we observed no evidence that the base frequency stimulation differed across the levels of the experimental factors.

#### Oddball (i.e., 1.25 Hz) response analysis

Repeated-measures (Bayesian) ANOVA with condition (2 levels), mixed format (3 levels) and electrode side (3 levels) as within-subject factors was performed.

#### Main effects

There was a main effect of condition, *F*(1, 18) = 39.41, *p* < 0.001, *η*_*p*_^2^ = 0.69, BF_Incl_ > 100, suggesting higher amplitudes for experimental than for control condition, *p*_bonf_ < 0.001, *d* = 1.44, BF_10_ > 100. There was no main effect of notation, *F*(2, 36) = 0.48, *p* = 0.62, *η*_*p*_^2^ = 0.03, BF_Incl_ < 1, and no main effect of electrode side, *F*(2, 36) = 0.92, *p* = 0.41, *η*_*p*_^2^ = 0.05, BF_Incl_ < 1.

#### First-order interactions

**Figure 4:**
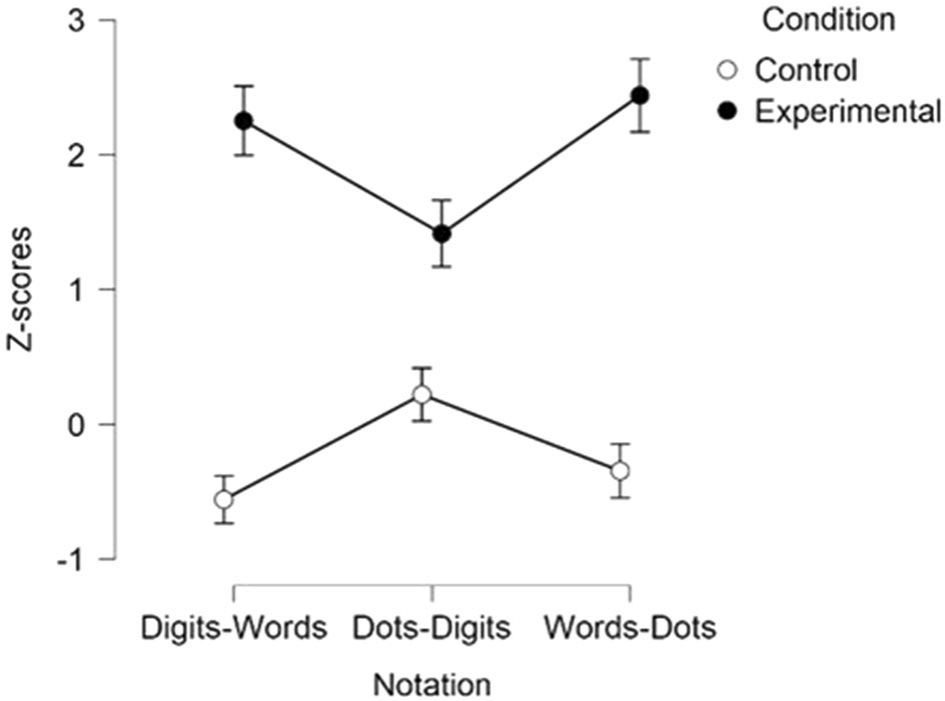
Z-scores for the mixed notation trials, depicted per condition across all electrode sides. Vertical bars denote Standard Errors (SE).

Although Bayesian ANOVA did not provide evidence for a condition × notation interaction, BF_Incl_ < 1, the classical results were significant, *F*(2, 36) = 5.73, *p* = 0.007, *η*_*p*_^2^ = 0.24 (see Fig. 4). In line with the results depicted in Table 1, post-hoc comparison (Bonferroni correction applied) showed that experimental conditions yielded higher oddball amplitudes than the control condition only for digits-words*, p*_bonf_ < 0.001, and words-dots, *p*_bonf_ < 0.001, notations, but not for digits—dots*, p*_bonf_ = 0.25. The strength of the amplitudes across digits—words and words—dots, did not differ from each other, either in the experimental, *p*_bonf_ = 1.00, or in the control conditions, *p*_bonf_ = 1.00. There was no condition × electrode side interaction, *F*(1.37, 24.69) = 0.37, *pGG* = 0.62, *η*_*p*_^2^ = 0.02, BF_Incl_ < 1, nor notation × electrode side interaction, *F*(4, 72) = 2.15, *p* = 0.084, *η*_*p*_^2^ = 0.11, BF_Incl_ < 1.

#### Second-order interactions

The three-way interaction between condition, notation, and electrode side was not significant, *F*(4,72) = 1.56, *p* = 0.194, *η*_*p*_^2^ = 0.08, BF_Incl_ < 1.

## Discussion

In the current study, we aimed to address the fundamental question about number abstraction by examining the automatic (i.e., unintentional) integration across numerical formats (i.e., digits, dots, and number words) using a frequency-tagged EEG technique.

Within the single notation trials, our results showed significant magnitude-related oddball responses over the whole posterior scalp in all experimental conditions but none in the control conditions. This indicates that participants were able to discriminate spontaneously and unintentionally between numbers, irrespectively of their notation. For mixed notations, the overall results showed significant oddball responses, indicating an unintentional magnitude abstraction across some notations: digits–words and words–dots, but not dots–digits. Consequently, the current study provides evidence that digits—words and words—dots are rapidly integrated to form an abstract representation of magnitude. However, in contrast to the findings of Liu et al.^23^, we found no evidence for an automatic integration between digits and dots.

In the three single notation conditions, we detected unintentional processing, but with higher amplitude for dots than words and digits. Concerning the dot format, our results extend previous studies on large numerosity discrimination^40–44^ to smaller numerosities, which, as demonstrated by the frequency-tagged EEG responses, highlights the unintentional nature of small numerosity processing (i.e., dots arrays ranging from 1 to 9). Concerning the symbolic format conditions (i.e., digits and words), our results replicate the findings reported by Guillaume, Poncin et al.^45^, showing that the magnitude of digits is automatically extracted. Finally, we also evidenced for the first time frequency-tagged EEG responses to the magnitude of number words.

When it comes to the lateralisation pattern of the single notation conditions, the presented results are in line with the hemispheric specialisation, typically reported in tasks involving different numerical formats. That is, significant oddball responses for the digits were obtained predominantly in the right posterior regions, while the words amplitude responses were mostly left-lateralised ^30,38,45,51,52^. The responses for the dots conditions were obtained across all posterior electrode sides, but were mostly centred along the posterior midline and right occipito-parietal regions^21,22,41,42,44^. These latter results possibly indicate the involvement of occipital regions along with parietal regions in numerosity extraction early in the visual stream (i.e., primary visual cortex^41,44^). On the other hand, despite the fast presentation pace (10 stimuli per second) the magnitude meaning carried by digits and number words seems to elicit exclusively higher-level parietal regions, with number words being more left-lateralised, as suggested by previous SSVEP studies involving words and letters^38,52^. This further implies that the unintentional magnitude extraction for symbolic numbers (digits and number words) seems to rely predominantly on higher-order processes and higher-level parietal brain areas, such as IPS, and language-related processing areas, such as the Visual Word Form Area (VWFA). In the case of dots, the magnitude extraction seems to happen earlier in the visual stream and involves both lower-level occipital areas as well as parietal areas^41,44^. Although we did not directly examine this question, the response pattern observed in the dots condition is generally in line with the claim, that the processing of the approximate numbers also involves the processing of the low-level non-numerical features (e.g., size, total area etc^53–56^). Overall, the observed difference between the levels of magnitude extraction possibly suggests that there is no automatised matching between a dot array and a number^57,58^, and is also broadly in line with the view that the symbolic and non-symbolic number processing relies on distinct neuro-cognitive mechanisms^21,22^.

Nevertheless, our results also showed that human adults are capable of integrating numbers of various formats (i.e., digits, number words, and dots), but the extent to which this process occurs unintentionally depends on the numerical formats (for similar claims see^59,60^). Concretely, our study provided evidence that such integration occurs automatically between number words and dots, and digits and number words, but we did not find conclusive evidence for an unintentional integration between digits and dots. The observation that oddballs were present for the mixed digits—words and dots—words, but not for the mixed digits—dots condition, possibly indicates that a direct cognitive link exists between both number words and digits, and number words and non-symbolic format. In contrast, the link between digits and non-symbolic format could occur indirectly. The presence of a direct link between digits and number words and number words and dots, but not between dots and digits, is further supported by the fact that presentation of less than 100 ms was sufficient to trigger oddball responses in the two former conditions. An indirect link between digits and non-symbolic numerosities could also explain why such integration was found in the study by Liu et al.^23^ but not in the current one. Concretely, in the current study, we used a very short stimulation (< 100 ms) thus capturing early cognitive processes and tapping into the very early stages of the numerical integration. While, Liu et al.^23^ observed some cross-format integration within ERP components appearing later in the processing stream, i.e., N1 (130–200 ms) and P2p (200–250 ms). It is, therefore, possible that due to their direct link, digits and number words, and dots and number words are integrated spontaneously and unintentionally and early in the processing stream, while digits and numerosities are integrated less rapidly and later in the processing stream, because of their indirect link. Put into perspective, such an indirect link seems to resemble the developmental trajectory of number format acquisition. Concretely, it has been demonstrated that children first learn to directly associate numerosities with number words and digits with number words, while the relation between digits and dots is acquired indirectly, through the children’s knowledge of how number words relate to both dots and digits^61–64^. Possibly, the trace of this acquisition pattern persists into adulthood as reflected by the pattern of results of the current study.

A limitation of the current study worth mentioning concerns the pure dot experimental condition. Within this condition, all standard stimuli were smaller than five, while the deviant stimuli were larger than five. However, as previous literature has demonstrated, small and large numbers are possibly processed in distinct perceptual systems^30^, where small numbers are subitized^65^, while larger numbers are approximated. An alternative view also exists, arguing that both small and large quantities are processed in one perceptual system^66,67^, where the differences in the representational precisions are possibly due to the allocation of /loading on additional cognitive resources (e.g., attention, working memory). Nevertheless, one could argue that the oddball amplitudes obtained in this condition were driven by participants switching between two perceptual systems, rather than unintentionally discriminating the magnitude. Based on our findings we cannot completely rule out this possibility, thus further studies will be needed in that direction. Nonetheless, the results from the dots condition were very similar to our previous studies^40–44^ where larger numerosities (e.g., 10 to 24 dots) were used.

In sum, the current findings show first, that the magnitude of numbers can be unintentionally processed under different notations (digits, number words, and numerosities); and second, that the magnitude can be abstracted, to some extent, across formats as evidenced by frequency-tagging EEG responses to magnitude changes in the two of the mixed notation conditions. The oddball responses found for digits, dots, and number words, indicate that numerical magnitude is rapidly extracted in both symbolic and non-symbolic numbers. Furthermore, our results suggest that frequency-tagged EEG can be used to track the integration between digits, number words, and dots. The current study highlighted such integration between digits and number words, and number words and dots. This pattern of results indicates that the way numbers are abstracted in the human adult mind seems to closely mirror the developmental pathway for numerical formats acquisition: a direct link of number words with non-symbolic numbers and digits but an indirect link between digits and numerosities.

## Supplementary Information

Supplementary Information.
