# Supplementary material for: Automatic integration of numerical formats examined with frequency-tagged EEG
Source: Sci Rep. 2021 Nov 1;11:21405. doi: 10.1038/s41598-021-00738-0 (PMC8560945; doi:10.1038/s41598-021-00738-0)
Supplement: Supplementary file 1 — Supplementary Information. [file 41598_2021_738_MOESM1_ESM.pdf]

Automatic Integration of Numerical Formats Examined with Frequency-tagged EEG

Mila Marinova[1,2,3], Carrie Georges[1], Mathieu Guillaume[4,5], Bert Reynvoet[2,3], Christine Schiltz*[1], and Amandine Van Rinsveld*[4,5]

[1] Institute of Cognitive Science and Assessment, Department of Behavioural and Cognitive Sciences, Faculty of Humanities, Education and Social Sciences, University of Luxembourg, Esch-Belval, Luxembourg

[2] Faculty of Psychology and Educational Sciences, KU Leuven @Kulak, Kortrijk Belgium.

[3] Brain and Cognition, KU Leuven, Leuven, Belgium

[4] Center for Research in Cognition and Neurosciences, ULB Neuroscience Institute, Université Libre de Bruxelles (ULB), B-1050 Bruxelles, Belgium

[5] Graduate School of Education, Stanford University, 505 Lasuen Mall, Stanford, CA 94305, USA

*Equal last authorship

Correspondence concerning this article should be addressed to Mila Marinova, Department of Behavioral and Cognitive Science, FHSE, University of Luxembourg Maison des Sciences Humaines 11, Porte des Sciences; L-4366 Esch-sur-Alzette, Tel: +32468219730; E-mail: mila.g.marinova@gmail.com

ORCID:

Mila Marinova: https://orcid.org/0000-0002-6875-7742

Carrie Georges : https://orcid.org/0000-0001-7492-7480

Mathieu Guillaume: https://orcid.org/0000-0002-6173-4687

Bert Reynvoet: https://orcid.org/0000-0002-4898-2475

Christine Schiltz: https://orcid.org/0000-0002-1055-3878

Amandine Van Rinsveld: https://orcid.org/0000-0001-7130-2469

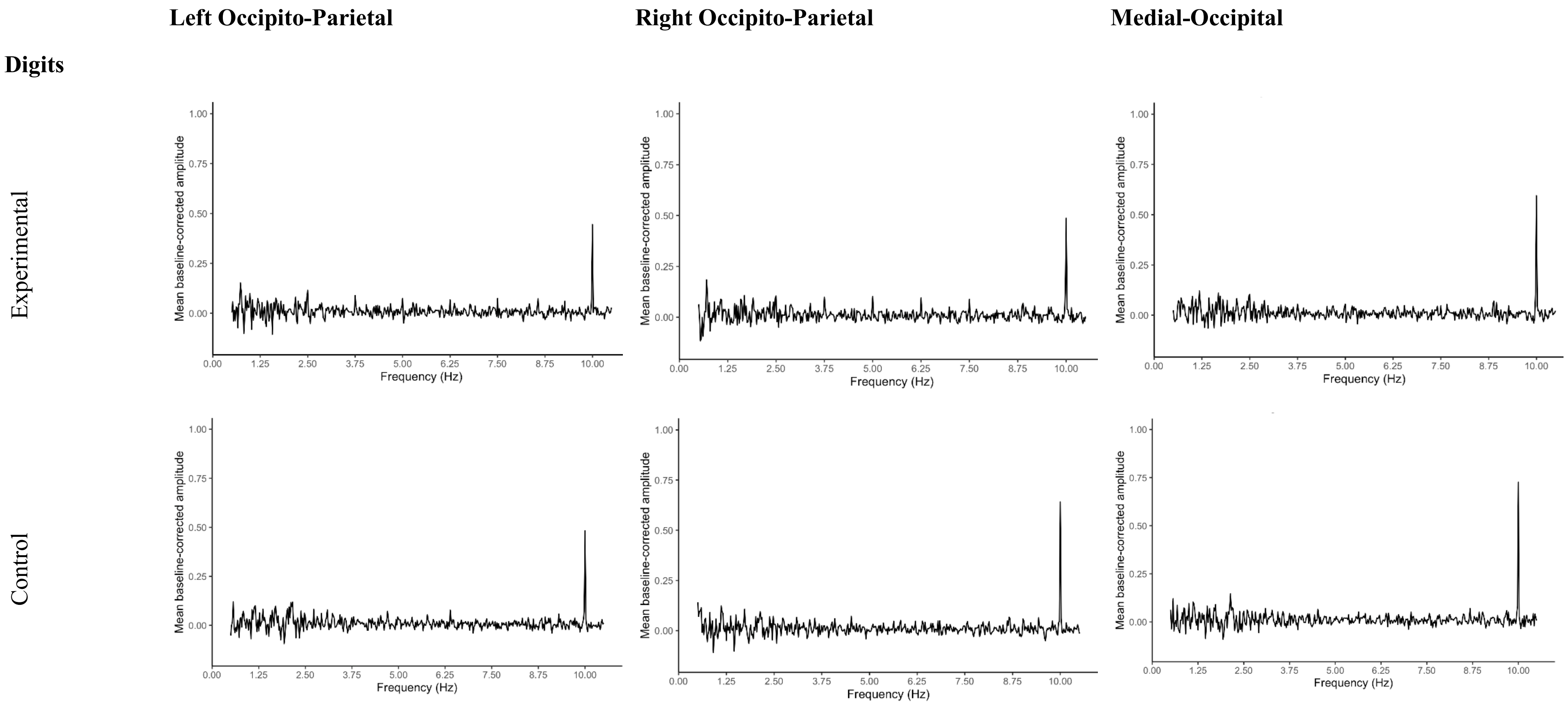
Left Occipito-Parietal
Right Occipito-Parietal
Medial-Occipital
Digits
Experimental
Control
Mean baseline-corrected amplitude
Frequency (Hz)
0.00
0.25
0.50
0.75
1.00
1.25
2.50
3.75
5.00
6.25
7.50
8.75
10.00

Words

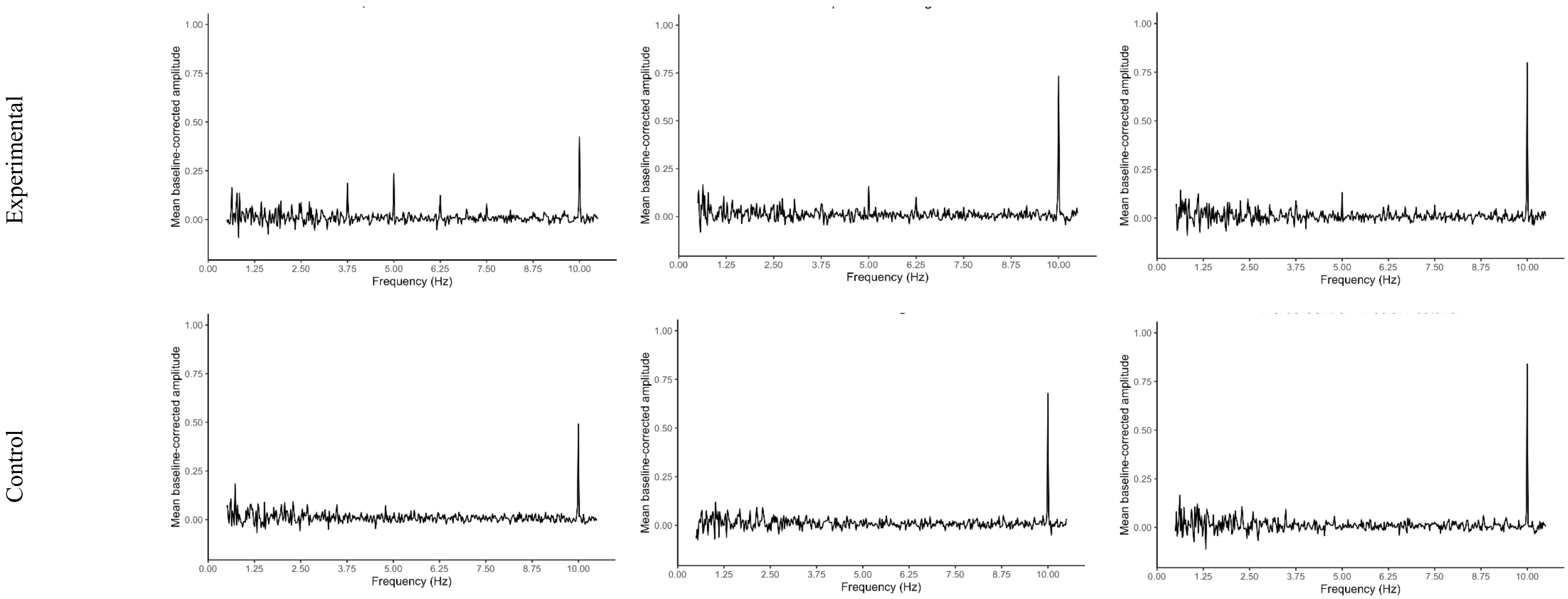
Experimental
Control
Mean baseline-corrected amplitude
1.00
0.75
0.50
0.25
0.00
Frequency (Hz)
0.00
1.25
2.50
3.75
5.00
6.25
7.50
8.75
10.00

Dots

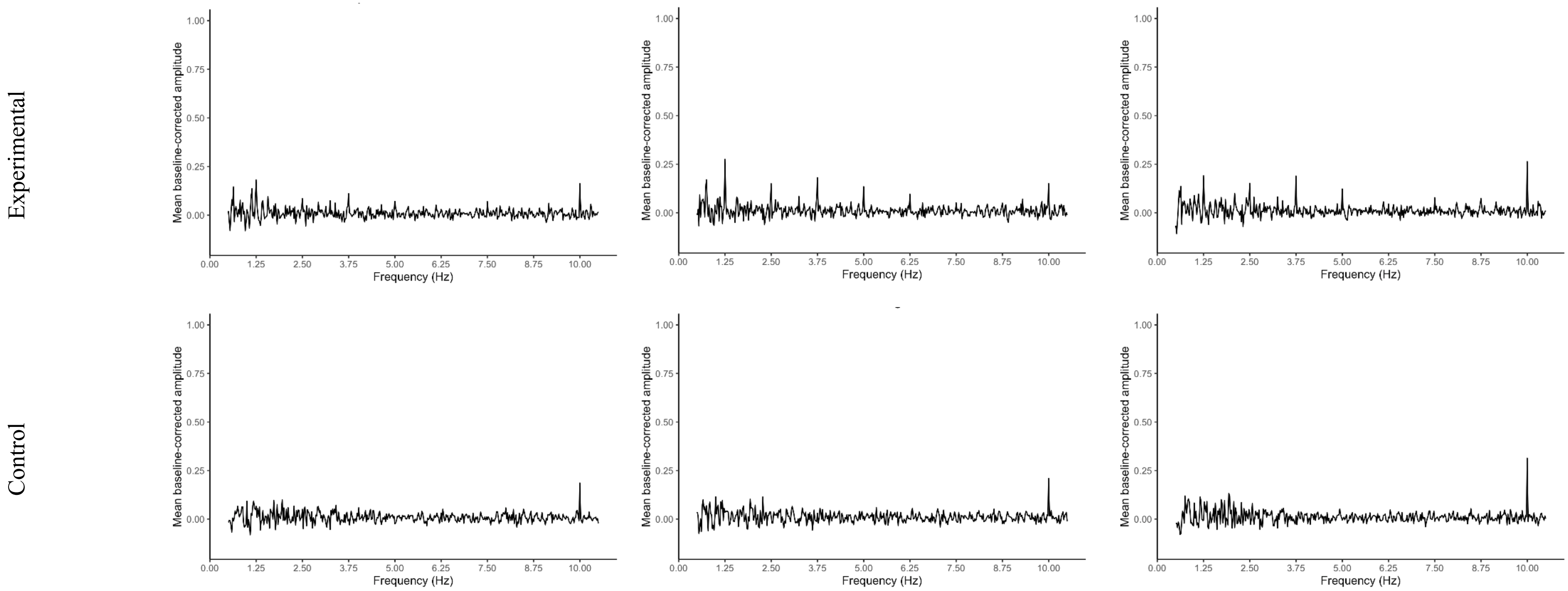
Experimental
Control
Mean baseline-corrected amplitude
Frequency (Hz)
0.00
0.25
0.50
0.75
1.00
0.00
1.25
2.50
3.75
5.00
6.25
7.50
8.75
10.00

Digits-Words

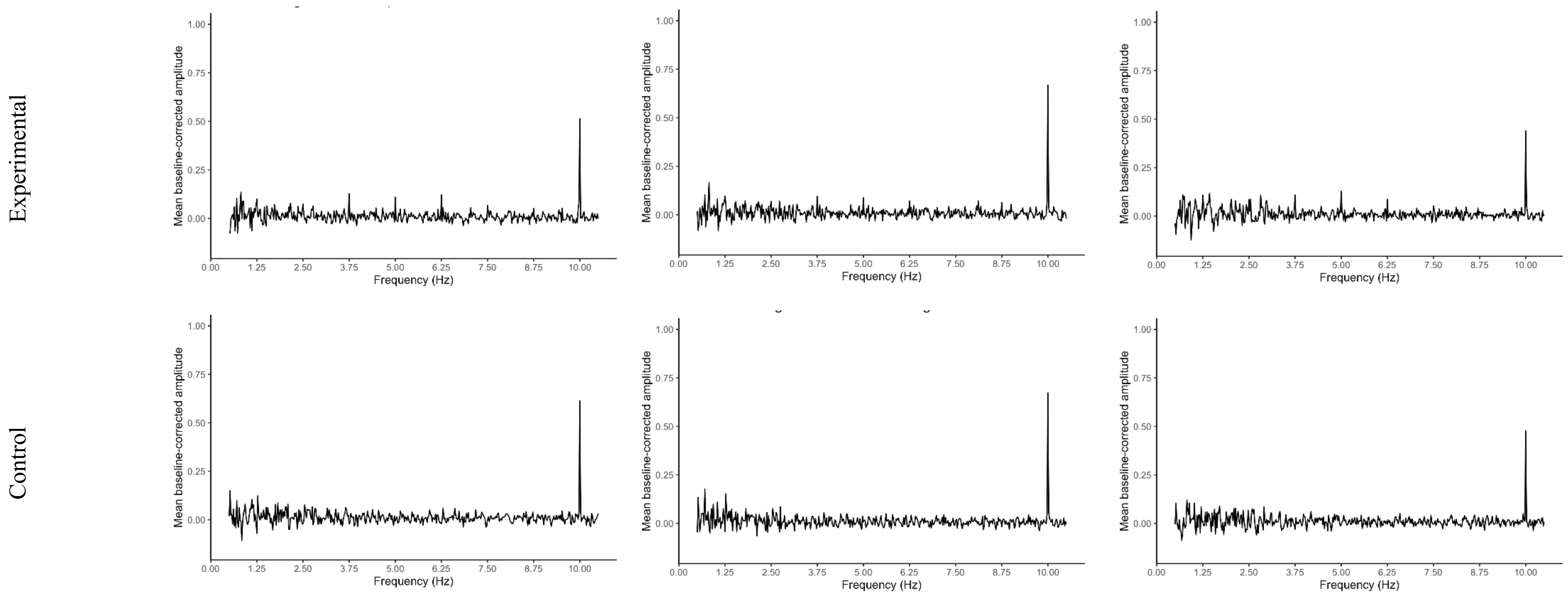

Dots-Digits

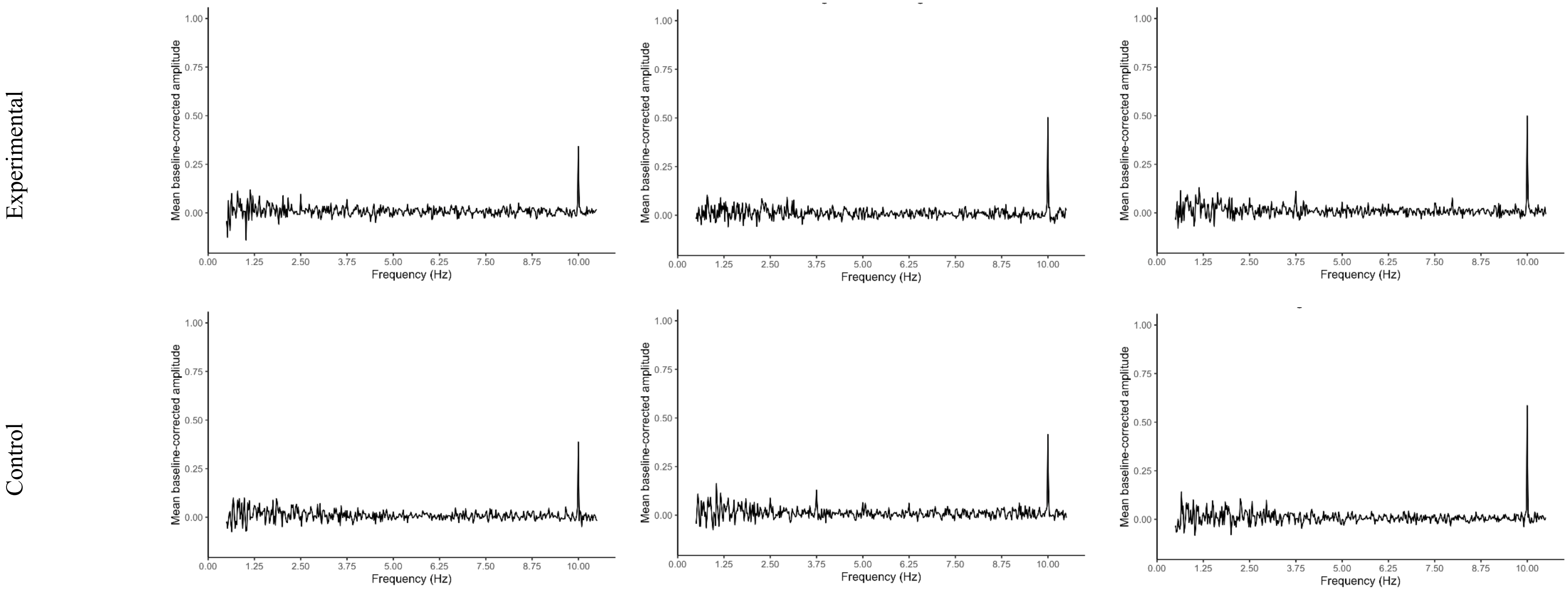
Experimental
Control
Mean baseline-corrected amplitude
Frequency (Hz)
1.00
0.75
0.50
0.25
0.00
0.00
1.25
2.50
3.75
5.00
6.25
7.50
8.75
10.00

Words-Dots

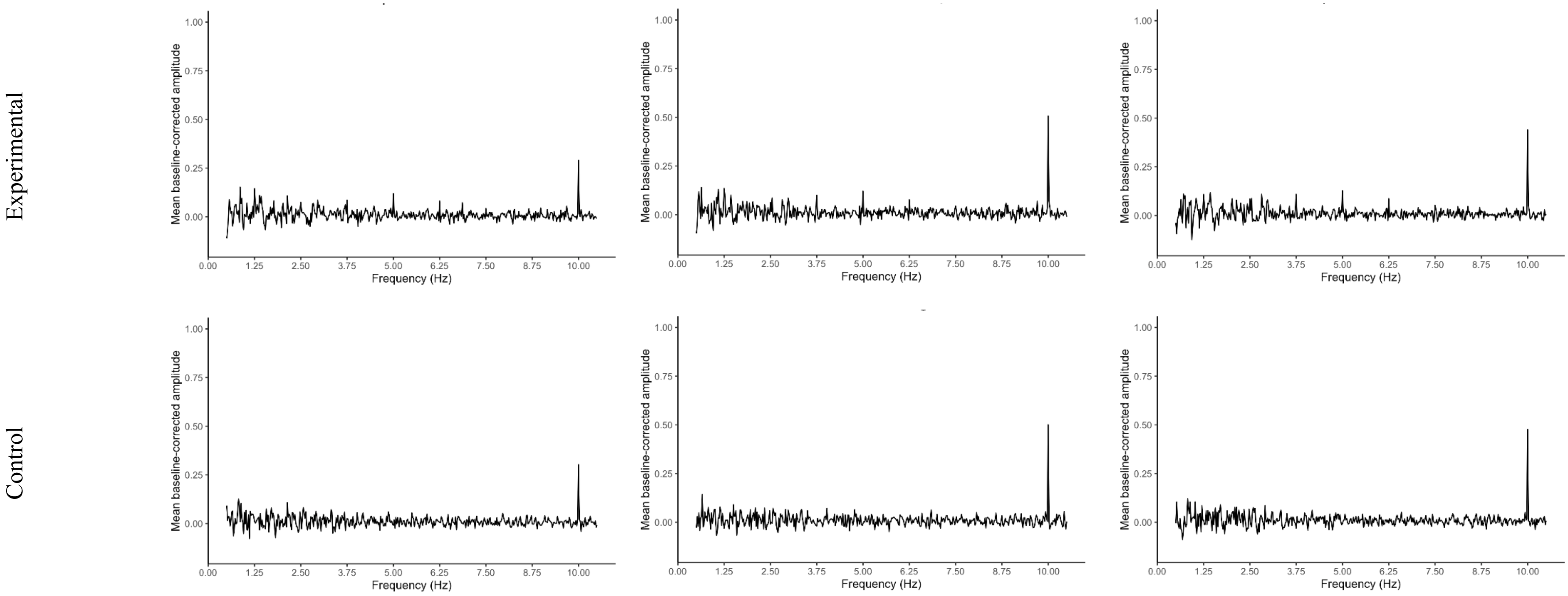

Figure S1.Amplitude spectra expressed as BA (baseline-corrected amplitudes) of the base frequency (10 Hz), oddball frequency (1.25Hz) and its seven harmonics, depicted per notation, condition, and electrode side.
